# Veterinary student involvement in research is driven by career aspirations and limited by time demands: An administrator survey study

**DOI:** 10.3389/fvets.2026.1810745

**Published:** 2026-05-19

**Authors:** Julie A. Hunt, Sarah Bolar, Ashutosh Verma

**Affiliations:** College of Veterinary Medicine, Lincoln Memorial University, Harrogate, TN, United States

**Keywords:** accreditation, research, research interest, student, survey, veterinary

## Abstract

**Introduction:**

The integration of research into veterinary education is vital and contributes to students’ competency in information literacy, scientific reasoning, and data interpretation, which are critical to generating high-quality patient care and public health outcomes. Although veterinary schools have implemented a range of initiatives to encourage student involvement in research, participation remains limited and inadequately quantified.

**Methods:**

A survey of administrators at veterinary schools accredited or provisionally accredited by the American Veterinary Medical Association’s Council on Education sought to quantify veterinary students’ involvement in research and elucidate perceived motivating factors and deterrents. The survey also investigated how research opportunities were valued by administrators, offered to students, and supported or rewarded. The survey also collected information about the adequacy of faculty available for mentoring and performing research and administrators’ desire for accreditor guidance about student involvement in research.

**Results:**

Administrators from 34 veterinary schools indicated that a median of approximately 20% of veterinary students at their institutions participated in research. Administrators perceived that students were motivated by career aspirations, enhancement of their internship or residency applications, and personal intellectual interest. Time, followed by students’ financial pressures, was thought to be the most significant barrier to student participation. Administrators reported variable perceived adequacy of faculty researchers and research mentors. Administrators were divided on whether additional accreditor guidance on research requirements and/or reporting would be helpful or harmful.

**Discussion:**

Median veterinary student participation in research was low at 20%. Veterinary schools may be able to increase student participation in research by promoting it as a means of career advancement, particularly for students who are interested in pursuing an internship or residency. Including research activities in the student academic timetable or maximizing research opportunities offered during academic breaks may also induce more student involvement.

## 1 Introduction

The integration of research into veterinary education has become increasingly vital in preparing future veterinarians for evidence-based clinical practice, innovation, and leadership in One Health initiatives. In a profession grounded in science and continually shaped by emerging knowledge, it is important that veterinary students not only consume scientific literature but also develop the skills to question, generate, and apply research and research knowledge. Research engagement fosters valuable competencies such as information literacy, scientific reasoning, and data interpretation, which are critical to delivering high-quality patient care and advancing public health outcomes (1). Students who participate in structured research experiences demonstrate stronger science identity, increased confidence in scientific discourse, and a greater willingness to participate in future research projects or pursue research-related careers (2–4). Accrediting bodies such as the American Veterinary Medical Association (AVMA) Council on Education (COE) have recognized incorporating research exposure as a key component of veterinary curricula (5).

Veterinary schools worldwide have implemented a range of initiatives to enhance students’ research engagement. In Australia, the University of Sydney embedded compulsory research projects into the veterinary curriculum (6). In the United Kingdom (UK), the University of Nottingham required all final-year students to complete a dissertation, while many UK programs offer intercalated Bachelor of Science degrees to facilitate focused research training (7). In the United States, research electives, for-credit seminar courses, and structured summer scholar programs provide early research exposure (8). Notable initiatives include the Boehringer Ingelheim Veterinary Scholars Program, the National Institute of Health-funded T35 summer fellowships, and the Cornell Leadership Program, all of which combine funding, mentorship, and scientific immersion to inspire student researchers (4, 9–11). Programs such as the Biomedical Research for University Students in Health Sciences (BRUSH), designed to support underrepresented students have further broadened the accessibility and appeal of research engagement to a diverse student body (3).

Despite these efforts, veterinary student involvement in research remains limited. The demanding and inflexible nature of veterinary curricula leaves little time for students to engage meaningfully in research during the academic year. Financial pressures and high educational debt disincentivize students from pursuing unpaid research roles, particularly when compared to clinical practice (12). Moreover, access to research mentors with well-resourced projects is uneven across institutions, especially in regions where research infrastructure is underdeveloped (13). Compounding these structural challenges are widespread misconceptions among students that research is less relevant, less rewarding, or incompatible with their goal of pursuing clinical practice. Consequently, while many students express appreciation for the value of research, relatively few pursue meaningful participation in it. Those students who have pursued intercalated research degree programs have described feelings of isolation or of being the “black sheep” among their peers (14). Ultimately, a cohesive, scalable approach to integrating research into global veterinary education remains elusive, and unsurprisingly, there remains a significant shortage of veterinarian-scientists in the United States (15, 16).

Comprehensive data on veterinary student engagement in research at AVMA COE-accredited institutions are lacking, limiting our ability to assess the extent of student participation during veterinary training, the factors motivating their involvement, and the barriers that discourage participation. This study aims to address this gap by (1) quantifying veterinary student participation in research across AVMA COE accredited veterinary colleges, (2) identifying the factors that encourage and support students’ research engagement, and (3) elucidating the primary challenges and deterrents that inhibit broader student involvement in research activities.

## 2 Methods

The study was reviewed and approved by the Institutional Review Board at Lincoln Memorial University (protocol #2025/04/18, approved May 14, 2025).

### 2.1 The survey

A Qualtrics survey was developed to gather information on veterinary students’ research involvement, trends in research topic interest, and existing structures for student participation. This survey was sent to administrators involved in developing research programs, specifically deans, associate or assistant deans of research, vice presidents of research, and directors of research. Surveys were voluntary and anonymous, although if administrators disclosed both their institution and their title, their identity could likely be discovered. The survey was 25 to 27 questions, depending on administrators’ answers, and was anticipated to take approximately 10 min to complete.

### 2.2 Students in research

The survey gathered background information such as which college the administrator worked at, the administrator’s role, yearly class size, percentage of the student cohort participating in research, and whether student participation was increasing or decreasing. To understand students’ participation in research at the college, the survey asked what research opportunities were available to students, what financial support was available to veterinary student researchers, what the administrators perceived motivated and inhibited student participation, how the institution promoted student involvement, and how the institution made students aware of research opportunities.

### 2.3 Faculty in research

To understand faculty availability, administrators were asked how many full-time equivalents (FTE) faculty were attributed to research, whether that was thought to be adequate, and if not, how many FTE would be sufficient. They were asked what percentage of faculty served as research mentors, whether that was thought to be adequate, and if not, how many would be sufficient.

### 2.4 Improving student engagement

Administrators were invited to leave open-ended comments on areas of improvement for student participation in research, on whether professional organizations or accrediting bodies could improve student participation in research, and whether the American Veterinary Medical Association Council on Education College on Education should set specific minimum expectations for student participation in research (Supplementary Appendix 1).

### 2.5 Survey dissemination

The surveys were reviewed, approved, and emailed by the American Association of Veterinary Medical Colleges (AAVMC) Educational Research Committee to Deans and Assistant/Associate Deans of Research at the 53 accredited veterinary schools and the 4 provisionally accredited veterinary schools internationally. Administrator surveys were emailed in August 2025. A single reminder email was sent out by the AAVMC approximately one month later.

### 2.6 Statistical methods

In the event that more than one survey was submitted for a single institution, the submission that was more complete was utilized for data analysis. If both submissions were complete, the submission from the administrator more likely to have a thorough knowledge of the research program was used (e.g., the Associate Dean of Research was considered likely to have the highest level of knowledge). If the same individual completed the survey twice, the survey that was completed on the earlier occasion was utilized for data analysis.

Likert-scale survey data were described using median. Continuous survey data were evaluated for normality using Shapiro–Wilk. Student enrollment at colleges and number of faculty full-time equivalents (FTEs) devoted to research were normally distributed and described using mean and standard deviation. All other data were non-normally distributed and were summarized using the median, interquartile range, and range. Proportion of faculty serving as research mentors were compared among groups using Kruskal-Wallis. A single author (JH) reviewed the open-ended survey data in Microsoft Excel and grouped comments that were similar; the frequency of each type of comment was reported in tables.

## 3 Results

### 3.1 Survey respondents

Forty-two responses were received from 34 unique veterinary schools, a 60% response rate (34/57 schools). Eight of those schools (8/34, 24%) submitted more than one survey response, either from both the Dean and the Assistant/Associate Dean, or from individuals to whom they forwarded the survey (e.g., Associate Dean of Academic Affairs, or Director of Summer Research Program).

Of the 34 veterinary schools, 25 were located in the United States, 4 were in Canada, 3 in Australia, one in the United Kingdom, and one in Puerto Rico. The person completing the survey was the Assistant/Associate Dean of Research at twenty-three colleges (23/34, 68%), the Dean at six colleges (6/34, 18%), the Associate Dean of Academic Affairs at two colleges (2/34, 6%), and another administrator at three colleges (3/34, 9%).

### 3.2 Students in research

Administrators reported that their colleges had a mean of 119.3 students per cohort (standard deviation 35.1, range 33–180). Most administrators reported that their colleges thought that promoting research skills among veterinary students was very important (25/34, 74%) or somewhat important (7/34, 21%); the remaining administrators reported that their colleges thought the value of research skills was neutral (2/34, 6%) or somewhat unimportant (1/34, 3%). Twenty-seven administrators (26/34, 76%) reported that their college reported tracking student participation in research, while 5 (5/34, 14%) were in the process of developing a tracking system, and 3 (3/34, 9%) stated that they did not track student participation in research. Administrators reported a median of 22.5% of the students at their college were involved in research (IQR 23.5%, range 10–100%). When data from the 5 administrators who reported that 100% of their students were involved in research were excluded (i.e., from colleges where participation was a mandatory part of the curriculum), then the median was 20% of the students were involved in research (IQR 17.0%, range 10–50%). There was no significant difference between participation rates at public and private institutions (median 20% public, 27% private including all responses; median 20% public, 18.5% private with 100% responses excluded).

Administrators described their colleges disseminating information about available research opportunities to students via email (31/34, 91%), the college’s website (30/34, 88%), face to face classes (25/34, 74%), student clubs (14/34, 41%), social media (13/34, 38%), newsletter (11/34, 32%), dean’s class/meetings (10/34, 29%), virtual learning environment (5/34, 15%), and at other events, including at orientation and research day. Thirty-three administrators (33/34, 97%) reported that their colleges offered either internal or external summer research scholar programs, while 30 administrators (30/34, 88%) reported informal faculty-student research collaborations occurring either during the summer or academic year. Twenty-four administrators (24/34, 71%) stated that their colleges offered a research elective course, and 20 administrators (20/34, 59%) said that students at their college were required to take a research course or had research projects as a required part of their curriculum. Eighteen administrators (18/34, 53%) reported that their colleges offered formal veterinary/PhD or similar dual degree programs, and four administrators (4/34, 12%) said that their colleges offered honors research tracks or certificates. Four administrators (4/34, 12%) stated that their colleges had other research offerings, including a veterinary scientist program, paid casual research work, lab-based work during the semester, and a dual degree program in development.

Administrators reported that their colleges offered structured financial support to veterinary student researchers, including paid stipends (30/34, 88%), funding for travel to conferences (30/34, 88%), research grants or fellowships (24/34, 71%), and tuition remission for research involvement (6/34, 18%). Administrators described students’ participation in research at their colleges as increasing (7/34, 21%), stable (22/34, 65%), decreasing (3/34, 9%), or highly variable year-to-year (2/34, 6%). Administrators stated that their colleges performed biomedical research (34/34, 100%), translational/One Health research (33/34, 97%), clinical research (32/34, 94%), public health/epidemiological research (32/34, 94%), wildlife/conservation medicine research (25/34, 74%), educational research (22/34, 65%), industry focused research and development (20/34, 59%), and other research (3/34, 9%). Administrators reported that students at their colleges were most interested in clinical research (mentioned by 27/34 colleges, 79%), followed by biomedical research (24/34, 71%) and public health/epidemiological research (19/34, 56%).

Administrators reported that they believed that students were most commonly motivated to participate due to career aspirations, enhancement of their internship or residency applications, and personal intellectual interest in the research projects (Table 1). Administrators reported that they believed that the most significant barrier to student participation in research was students’ heavy curriculum and time demands, followed by students’ financial pressures or debt concerns and limited student interest (Table 1). Administrators reported that their colleges utilized several strategies for promoting student engagement in research, including early exposure to research (28/34, 82%); support for conference participation (26/34, 76%); research skills workshops or seminars (23/34, 68%); dedicated research mentorship programs (22/34, 65%); incentives such as awards, certificates, or transcript notations (18/34, 53%), and outreach to underrepresented student groups (9/34, 26%).

**Table 1:** Veterinary school administrators’ perception of factors that motivate or hinder student participation in research.

Question	Response	*N*, %
What motivates students at your college to engage in research? (Select the top 3)	Career aspirations (e.g., academia, research)	26/34, 76%
	Enhance their internship or residency application	22/34, 65%
	Personal intellectual interest or for interesting projects	22/34, 65%
	Financial incentives (e.g., stipends, scholarships)	13/34, 38%
	Mentorship or influence of faculty	12/34, 35%
	Required curricular component	4/34, 12%
	Other (career exploration)	1/34, 3%
What are the most common barriers that limit veterinary students’ participation in research at your institution? (Select all that apply)	Heavy curriculum demands or time constraints	26/34, 76%
	Financial pressures or debt concerns	14/34, 41%
	Limited student interest	14/34, 41%
	Limited number of available mentors or projects	10/34, 29%
	Lack of awareness of opportunities	9/34, 26%
	Structural challenges (scheduling, administrative hurdles)	9/34, 26%
	Other (summer clinical opportunities, lack of research experience, lack of funding, research wages being less than wages students can obtain in other summer jobs, or no barriers)	8/34, 24%

Fourteen administrators (14/34, 41%) reported that their college had a system in place to recognize students who completed significant research; they described a variety of recognitions including research awards, scholar certificates, monetary compensation, honor societies, course credit, intercalated degrees, research scholar designations, micro credential badges, and paper authorship. Several administrators (3/34, 9%) stated that they were aware that their college was currently in the process of developing a system to recognize veterinary student research effort. Administrators at 17 colleges (17/34, 50%) stated that their college did not offer any formal tracking or recognition for veterinary students who completed significant research activities.

### 3.3 Faculty in research

Administrators at 31 schools reported a range of 3–81 full-time equivalent (FTE) faculty devoted to research at their college (median 40, IQR 28); three administrators did not report the number of faculty FTEs devoted to research. Twenty-four administrators (24/34, 71%) stated that they thought the number of faculty FTEs devoted to research at their school was sufficient for interested students to have access to research opportunities, while 6 administrators said it may be sufficient (6/34, 18%), and four administrators said that it was not sufficient (4/34, 12%). Administrators who stated that they had sufficient research FTEs had a median of 41 FTEs (range 18–81) at their institutions. Those who felt they may have sufficient research FTEs had a median of 33 (20–71), whereas administrators reporting insufficient research FTEs had a markedly lower median of 3 (3–30). When the four administrators who answered that they did not have sufficient research FTEs were asked how many additional research FTEs would be needed to meet the student need, they responded with increases of 15–233% (median 133.5%, IQR 196.75) faculty research FTEs.

Administrators reported that 8–100% of the faculty (median 30.5, IQR 22.75) at their college mentored veterinary student researchers. Eighteen administrators (18/34, 53%) thought that this was a sufficient proportion, 7 administrators said that there may be a sufficient number of research mentors (7/34, 21%), 8 (8/34, 24%) said that it was not, and 1 (1/34, 3%) did not answer the question. Administrators stating that the proportion of faculty serving as research mentors was sufficient reported a median of 40% of their faculty serving as mentors (range 20–100%). Those reporting that there may be a sufficient number indicated a median of 20% of their faculty serving as mentors (8–65%), and those stating that an insufficient number of faculty served as research mentors reported a median of 18% (range 10–30%). The 8 administrators who said that there were insufficient research mentors stated that the number of research mentors would need to be increased by 14–150% (median 85%, IQR 77.75).

### 3.4 Improving student engagement

Administrators described a variety of ways veterinary students have successfully engaged in research at their colleges, most commonly mentioning summer research programs (Table 2). One administrator reported expanding their summer research program to include incoming first year veterinary students, while another administrator reported making their program more appealing by allowing students to end the summer with two weeks of optional clinical shadowing in the veterinary teaching hospital while students wrap up their research and prepare their posters. Several administrators also mentioned success with instituting mandatory student research programming, such as student research days or seminar series. Other administrators described seeing successful student research outcomes, including students co-authoring publications, presenting at professional conferences, and entering research careers upon graduation.

**Table 2:** Approaches employed by veterinary school administrators to engage students in research.

Method	Number of administrators reporting this
Offering summer research student programs; one administrator described offering this program to incoming veterinary students	9
Engaging students in mandatory research programming (e.g., student research day, research seminar series, bimonthly meetings)	4
Sharing student research work outputs (e.g., students co-authoring publications, presenting at professional conferences, entering research careers after graduation)	4
Mandating that students complete a research course	3
Engaging students in dual degree programs	2
Providing funding for students to go to developing countries to perform field research	1
Creating a One Health program and establishing a veterinarian scientist program	1
Providing extra skills training in communication and scientific writing to all research students	1
Mixing veterinary students, house officers, and PhD students together for research work	1

This data was reported by 21/34 (62%) of survey respondents; some respondents reported multiple approaches.

Administrators offered a variety of suggestions for enhancing veterinary students’ involvement in research at their colleges; the most common recommendations were to increase research funding, increase student research stipends, and integrate research into students’ curriculum (Table 3).

**Table 3:** Resources and changes recommended by administrators to enhance student involvement in research.

Recommendation	Number of administrators recommending this
Find additional research funding (e.g., from extramural sources, for additional projects, for additional summer research students, for PhD students)	12
Increase student research stipends	6
Integrate research into the veterinary curriculum	5
Improve communication of research opportunities to students; improve system to connect students to faculty running research	2
Find room in the curriculum for students to do research	2
Offer more diverse research topics, including clinical research	2
Have more research mentors and projects	2
Increase student awareness of research opportunities outside of summer research training program	1
Offer virtual or hybrid virtual research opportunities	1
Assure students of rewarding/fulfilling career in academia bolstered by research	1
Add or streamline research electives during clinical year	1
If using clinical examples in lectures, include unknowns that need to be discovered to improve clinical outcomes	1
Develop a career plan for students showing interest in research and emphasize this pathway during admissions interview	1
Develop a dual degree program	1
Add access to agricultural animal BSL2 facilities	1
Offer additional funds available for conference travel	1
Seek more emphasis on research from central administration	1
Seek more value placed on research competencies by AVMA standards	1

This data was reported by 33/34 (97%) of survey respondents; some respondents reported multiple recommendations.

### 3.5 Professional organizations and accrediting bodies

Administrators described several ideas of actions that professional organizations and accrediting bodies could take to improve student participation, including offering financial support to students who engage in research and maintaining high standards for research for accreditation (Table 4). One administrator called on accrediting bodies to specifically define how the research standard and competencies could be met. Another administrator suggested that students’ competency in research should be equivalent to their clinical competency.

**Table 4:** Administrators’ opinions about what professional organizations or accrediting bodies can do to improve student participation in research.

Recommendation	Number of administrators recommending this
Offer financial support to engage students in research (e.g., for research, for travel to conferences, competitive summer scholarships, for Veterinary Research Scholars Program, dual degree programs)	11
Maintain high standards for research for accreditation. The current standard is low (e.g., does not define where/how research competencies are met). Make exposition of real research at their college mandatory for accreditation. Consider making students’ competency in research have to be similarly strong as their clinical competency.	4
Provide externally funded research programs; provide better funding for clinical research; small grant support for faculty who are willing to mentor students but lack competitiveness for large extramural funds;	3
Support programs for students to present their work	2
Provide mechanisms for faculty to devote more time to student involvement in research or providing research mentorship	2
Help with faculty recruitment strategies for research-intensive faculty	1
Provide a system for connecting students to researchers	1
Provide training to faculty in how to coach and provide feedback to students in a workplace setting	1
Do not mandate	1
Introduce students to research careers outside academia	1
Do a comprehensive and inclusive analysis of the state of research in veterinary colleges, including trends, opportunities, and challenges, and share the outcomes with all.	1
Create an expectation of a research project to address clinically relevant projects.	1
Provide career role modeling and exemplars for research careers	1
Fight to protect animal models when needed in research	1

This data was reported by 22/34 (65%) of survey respondents; some respondents offered multiple recommendations.

When administrators were asked whether the American Veterinary Medical Association’s Council on Education (AVMA COE), the accrediting body for veterinary schools in the United States, should set a minimum expectation for students’ participation in research, administrators were equally divided, with 13 administrators (13/34, 38%) commenting that they would like the research standard to provide minimum guidelines for students’ participation in research and 13 administrators (13/34, 38%) stating that they would not like the research standard to be prescriptive. Five administrators (5/18, 15%) purposefully abstained from offering an opinion, and 3 (3/18, 9%) provided comments that were ambiguous. The most common responses from the administrators were that the standard should require mandatory exposure to research topics in the curriculum (e.g., a mandatory research course), that the standard should require students to graduate with defined minimum skills in research (e.g., information retrieval, experimental design, etc.), and that no, there did not need to be any specified expectations for students’ involvement in research because the current language in Standard 10 (Research Programs) was adequate (Table 5).

**Table 5:** Administrators’ opinion on the need, or lack thereof, for the American Veterinary Medical Association’s Council on Education (AVMA COE) to include minimum expectations for students’ participation in research.

Recommendation	Number of administrators recommending this
Yes, they should recommend mandatory exposure in the curriculum (e.g., a research course)	4
Yes, they need some minimum skills (e.g., information retrieval, experimental design, science communication, research methods, evidence-based medicine)	4
No, the current standard 10 (research standard) is fine	4
No, it should be determined by all stakeholders (college, student, industry, government); we should get them together and figure out what research competencies are desired and teach to those	3
Yes, there should be class time for research; each student should have to do a research project during/for class	2
Yes, the current standard 10 is not specific enough, and it needs to be more objective in its expectations	2
No, colleges differ and a uniform prescribed standard is not appropriate; colleges should set their own standard	2
Yes, they should recommend better integration of DVM and graduate/postgraduate students	1
Yes, they should require a minimum number of faculty active in research	1
Yes, this would encourage the entire school (faculty and staff) to have some involvement in research	1
Yes, there should be prescribed standards but only for summer research programs	1
Yes, but the only requirement should be that if a student wants to participate in research, they can	1
No, students already have too much to do	1
No, the COE keeps adding rules and there are already too many	1

This data was reported by 28/34 (82%) of survey respondents.

## 4 Discussion

The strong response rate observed in this study suggests that student engagement in research is a topic of high priority to senior leadership at AVMA-accredited colleges of veterinary medicine. Nearly all respondents indicated that promoting student research was important, aligning with COE accreditation standards. Additionally, this suggests a shared recognition that research participation contributes to the development of critical thinking, scientific literacy, and professional identity formation in veterinary students.

Administrators reported that a median of 20% of students participated in research activities at their institutions. This figure has not been previously documented in the veterinary education literature and thus offers a valuable reference point. Institutions reporting universal participation were those that embedded research as a required curricular component. This approach ensures broad exposure, though its feasibility may vary across programs depending on curricular structure and time constraints. For institutions that do not require research participation, student involvement varied considerably, highlighting the role of institutional context in shaping student research experiences. Differences in faculty mentor availability, protected research time, and funding for student stipends likely contributed to this variability (17). Despite these differences, most institutions reported stable or increasing participation, suggesting that students are accessing and pursuing available opportunities. Although all responding institutions offered and promoted research opportunities, a median of only about 20% of students participated. This level of participation may influence faculty workload distribution and how costs of supporting research opportunities are absorbed within tuition-dependent educational models.

The modes through which institutions disseminate information about research opportunities reflect evolving educational and workplace norms. Virtual communication, primarily through email and institutional websites, was the most common strategy reported, consistent with the preferences of current learners and the professional environments they are preparing to enter (18). Nearly all institutions offered summer research programs, which were widely perceived as successful. These programs may be effective because they offer immersive experiences outside the academic year and typically include stipends to cover summer living expenses. In addition, more than half of institutions reported offering mandatory research courses, potentially as a mechanism for meeting accreditation expectations while promoting foundational research competencies. Research electives and dual-degree pathways were also common, illustrating the range of curricular strategies institutions employ to support diverse student interests and career trajectories.

Administrators perceived student motivation for research participation as primarily driven by long-term educational and career goals, including interest in research-intensive careers, academic pathways, and strengthening professional credentials. This is similar to research findings from Doctor of Medicine (MD) students, who reported that their motivation to participate in research was driven by a desire for academic and career advancement (19). However, a direct comparison is limited because this study’s veterinary data reflect administrator perceptions rather than student reports. Administrators perceived that students were also motivated by a desire to improve their internship or residency applications. Students’ focus on their career trajectory may have influenced the type of research that they elected to perform, with administrators reporting that clinical research was the most desired by students. Studies in human medical training also demonstrated that research experience as a medical student does improve the likelihood of matching for competitive residency programs (20–22). Veterinary residency programs reported that research experience and/or published research ranked behind only class rank and veterinary school grades as important to their selection process (23). One approach to increasing veterinary student involvement in research is to emphasize, early in their veterinary education, how research experience can strengthen internship and residency applications.

Despite the availability of research student stipends and fellowships at many institutions, administrators did not perceive that financial compensation was a primary motivator for student participation, which is an interesting finding since many students carry substantial educational debt. It is possible that students either do not view research stipends as attractive enough or are not aware that there is financial compensation for participating in research. However, it is also possible that students have not felt comfortable disclosing their financial situation to the administrators responding to this survey. Future research surveying participants and non-participants could clarify whether financial compensation was a primary motivator.

Administrators perceived limited time was students’ main barrier, reflecting the intensity and demands of veterinary training. This matches findings that indicated that limited time was a major factor discouraging MD students from participating in research (24). Concerns about student finances also emerged in the administrators’ survey, particularly considering the educational debt carried by many veterinary students. From an educational perspective, these findings suggest that students may view research participation as an enrichment activity rather than an integral component of professional training. Schools may be able to improve student participation in research by including research activities in students’ academic timetable or maximizing research opportunities offered during academic breaks.

Faculty capacity was generally viewed by administrators as sufficient to support student research engagement, although notable exceptions highlight persistent disparities among institutions and potential challenges in funding and/or hiring. There was considerable overlap in the number of FTEs reported between groups indicating sufficient, possibly sufficient, or insufficient research FTEs, suggesting that the research FTEs required to run a successful research program may be influenced by institutional factors. However, none of the respondents indicated sufficient or possibly sufficient researchers with fewer than 18 research FTEs. Likewise, there was considerable overlap in the proportion of faculty serving as research mentors between groups reporting sufficient, possibly sufficient, or insufficient number of research mentors, but there was no clear minimum value ensuring adequacy. Open-ended responses emphasized the importance of extramural funding in sustaining student research opportunities, particularly for stipend support and project resources. Administrators also reported diverse views regarding possible revisions to COE Standard 10 (Research Programs), with interest in strengthening student research competence alongside a desire to avoid overly prescriptive approaches. These views underscore a broader educational aim of fostering accountability while maintaining flexibility for institutions to tailor research experiences that align with their educational model, resources, curricular structures, and student needs.

Several limitations of our study should be acknowledged. Because this study focused on AVMA-accredited and provisionally accredited institutions, the findings primarily reflect a U.S.-centric educational context, despite approximately a quarter of responses coming from non-U.S. institutions. Assistant and Associate Deans of Research were the most common respondents; their focus on research likely impacted their assessment of the importance of research in veterinary education. Additionally, administrator perceptions of student motivations and barriers may not fully capture student perspectives. Future research incorporating direct student input, including qualitative data, is needed to better understand how students perceive and assess research and to guide integration into veterinary education. Finally, this study did not evaluate how inequity in research opportunities impacted students’ participation. Additional research is needed to understand whether student factors such as race, ethnicity, gender, and socioeconomic status may have influenced their involvement or non-involvement in research and thus impacted the 20–23% median participation rate.

## Data Availability

The anonymized raw data supporting the conclusions of this article will be made available by the authors upon request, without undue reservation.

## Supplementary material

Supplementary Datasheet 1 The administrator survey.

## References

[1] Janicke H Johnson M Baillie S Warman S Stone D Paparo S . Creating the next generation of evidence-based veterinary practitioners and researchers: what are the options for globally diverse veterinary curricula? J Vet Med Educ. (2020) 47:647–58. doi: 10.3138/jvme.2019-0098, 33231517

[2] Allan FJ Dunning MD Parkinson TJ Baillie S. Survey of 252 veterinary students to assess how undertaking a research project affects attitudes toward research in practice. J Vet Med Educ. (2021) 48:599–609. doi: 10.3138/jvme-2019-0151 33226901

[3] Ewart S Maves B Latona O Young L Sawtelle V Watts S . BRUSH summer research program: promoting science identity in underrepresented veterinary and undergraduate students. J Vet Med Educ. (2021) 48:599–609. doi: 10.3138/jvme-2024-0045 39504223 PMC11848847

[4] Fraser D McGregor D Grohn Y. Career paths of alumni of the Cornell leadership program for veterinary students. Vet Rec. (2008) 163:750–6. doi: 10.1136/vr.163.25.750 19103620 PMC4016953

[5] AVMA Council on Education. Accreditation Policies and Procedures of the AVMA Council on Education. (2025). Schaumburg, IL: American Veterinary Medical Association. Available online at: https://www.avma.org/sites/default/files/2025-05/coe-pp-Jan-2025_revised-Mar-2025.pdf#:~:text=The%20most%20recently%20updated%20version,the%20next%20scheduled%20review%20are (Accessed February 13, 2026).

[6] Slapeta J Ward M. Embedding research and enquiry in the Australian DVM curriculum. Aust Vet J. (2024) 102:324–8. doi: 10.1111/avj.13334, 38653562

[7] Royal Veterinary College. Bachelor of Veterinary Medicine with an Intercalated Year. (2014). London, UK: Royal Veterinary College. Available online at: https://www.rvc.ac.uk/study/undergraduate/bachelor-of-veterinary-medicine-intercalated (Accessed January 21, 2026).

[8] Chiu E Goldsmith E Moon C VandeWoude S. A model course to enhance veterinary student exposure to research. J Vet Med Educ. (2020) 47:445–51. doi: 10.3138/jvme.0718-081r, 31721645

[9] Boehringer Ingelheim. The Veterinary Scholars Program. (2019). Duluth, GA: Boehringer Ingelheim. Available online at: https://veterinaryscholars.boehringer-ingelheim.com/ (Accessed January 21, 2026).

[10] National Institutes of Health. NRSA Short-Term Research Training (T35). (2024). Bethesda, MD: National Institutes of Health. Available online at: https://grants.nih.gov/funding/activity-codes/T35 (Accessed January 21, 2026).

[11] McGregor D Fraser D. The Cornell leadership program for veterinary students. J Vet Med Educ. (2002) 29:157–61. doi: 10.3138/jvme.29.3.157, 12378433

[12] National Institutes of Health. Physician-Scientist Workforce Working Group Report. (2014). National Institutes of Health: Bethesda, MD. Available online at: https://acd.od.nih.gov/documents/reports/PSW_Report_ACD_06042014.pdf 10.1111/cts.12209 PMC5439807 25123835

[13] National Academies of Sciences Engineering, Medicine. Workforce Needs in Veterinary Medicine. Washington, DC: The National Academies Press (2013).

[14] Atchison M. Factors that attract veterinarians to or discourage them from research careers: a program director’s perspective. J Vet Med Educ. (2009) 36:76–82. doi: 10.3138/jvme.36.1.76, 19435993

[15] National Research Council. Animal Health at the Crossroads: Preventing, Detecting, and Diagnosing Animal Diseases [Internet]. Washington, DC: National Academies Press (2005).

[16] National Research Council. Critical Needs for Research in Veterinary Science. Washington, DC: National Academies Press (2005). 20669456

[17] Kogan LR Hellyer P Ruch-Gallie R Rishniw M Schoenfeld-Tacher R. Veterinarians’ use and perceptions of information and communication technologies. Med Res Arch. (2016) 4:1–29. Available online at: https://esmed.org/MRA/mra/article/view/502

[18] Chang Y Ramnanan C. A review of literature on medical students and scholarly research: experiences, attitudes, and outcome. Acad Med. (2015) 90:1162–73. doi: 10.1097/ACM.0000000000000702 25853690

[19] Beres A Baird R Puligandla P. Success in the pediatric surgery match: a survey of the 2010 applicant pool. J Pediatr Surg. (2011) 46:957–61. doi: 10.1016/j.jpedsurg.2011.02.030, 21616260

[20] Claiborne J Crantford J Swett K David L. The plastic surgery match: predicting success and improving the process. Ann Plast Surg. (2013) 70:698–703. doi: 10.1097/SAP.0b013e31828587d3, 23673567

[21] Rinard J Mahabir R Shenoy A Mahabir R. Successfully matching into surgical specialties: an analysis of national resident matching program data. J Grad Med Educ. (2010) 2:316–21. doi: 10.4300/JGME-D-09-00020.1, 21976075 PMC2951766

[22] Yanke A Shaver S Diehl K Woolcock A Lyon S Hofmeister E. Resident selection criteria in veterinary medicine. J Vet Med Educ. (2023) 51:376–83. doi: 10.3138/jvme-2022-0111, 37384579

[23] Siemens D Punnen S Wong J Kanji N. A survey on the attitudes towards research in medical school. BMC Med Educ. (2010) 10:1–7. doi: 10.1186/1472-6920-10-4 20096112 PMC2823602

