# Supplementary material for: Veterinary student involvement in research is driven by career aspirations and limited by time demands: An administrator survey study
Source: Front Vet Sci. 2026 May 19;13:1810745. doi: 10.3389/fvets.2026.1810745 (PMC13226102; doi:10.3389/fvets.2026.1810745)
Supplement: Supplementary Datasheet 1 — The administrator survey. [file Data_sheet_1.pdf]

**Demographics:**

1. What is the name of your veterinary college or institution: (Open-ended)
2. What is your role at your veterinary college?
    - Dean
    - Associate or Assistant Dean of Research
    - Vice President of Research
    - Director of Research
    - Other Position (please specify)
3. What is the approximate number of veterinary students enrolled per class year: (Numeric input)

**Student Research Involvement Metrics**

4. Does your college formally track veterinary student participation in research activities?
    - Yes
    - No
    - In development
5. Approximately what percentage of veterinary students at your college participate in research during their veterinary education? (Use slider to choose an answer)
6. What types of research involvement opportunities are available to veterinary students at your institution? (Select all that apply)
    - Summer research scholar programs (internal or external)
    - Curriculum-integrated research course or projects (required part of curriculum)
    - Elective research courses
    - Formal DVM/PhD or similar dual-degree programs (e.g., receive a research degree while earning a veterinary degree)
    - Honors research tracks or certificates
    - Informal faculty-student research collaborations
    - Other (please specify)
7. What structured financial supports are available for veterinary student researchers? (Select all that apply)
    - Paid stipends
    - Research grants or fellowships
    - Tuition remission for research involvement
    - Funding for travel to conferences
    - No specific financial support

**Observations on Student Behavior and Interest**

8. Based on your observations, how would you describe overall veterinary students' interest in research over the past 5 years?
    - Increasing
    - Decreasing
    - Stable
    - Highly variable year-to-year
9. Which research areas are currently offered by the faculty at your institution? (Select all that apply)
    - Clinical research
    - Biomedical laboratory research
    - Public health/epidemiology
    - Educational research
    - Wildlife/conservation medicine
    - Translational/One Health research
    - Industry-focused research & development
    - Other (please specify)
10. Which areas of research are most popular among interested veterinary students at your institution? (Select top three)
    - Clinical research
    - Biomedical laboratory research
    - Public health/epidemiology
    - Educational research
    - Wildlife/conservation medicine
    - Translational/One Health research
    - Industry-focused R&D
    - Other (please specify)
11. In your opinion, what motivates veterinary students at your college to engage in research? (Select top three)
    - Career aspirations (e.g., academia, research)
    - Enhancing residency/internship applications
    - Personal intellectual interest/interesting projects
    - Mentorship/influence of faculty
    - Financial incentives (stipends, scholarships)
    - Required curricular components
    - Altruistic desire to contribute to science

- Other (please specify)

12. What are the most common barriers that limit veterinary students' participation in research at your institution? (Select all that apply)
    - Heavy curriculum demands/time constraints
    - Financial pressures/debt concerns
    - Lack of awareness of opportunities
    - Limited number of available mentors/projects
    - Limited student interest
    - Structural challenges (scheduling, administrative hurdles)
    - Other (please specify)

**Institutional Strategies and Support**

13. Which of the following strategies does your institution use to promote veterinary students' engagement in research? (Select all that apply)
    - Dedicated research mentorship programs
    - Research skills workshops/seminars
    - Early exposure (first-year research orientation or courses)
    - Incentives such as awards, certificates, or transcript notations
    - Conference participation support
    - Outreach to underrepresented student groups
    - Other (please specify)
14. Does your college offer any formal tracking or recognition for veterinary students who complete significant research activities?
    - Yes (e.g., research honors, certificate, transcript notation) (Please specify)
    - No
    - In development
15. How does your institution inform veterinary students about research? (Select all that apply)
    - Email
    - Newsletter
    - College website
    - Student clubs
    - Socia media
    - Dean's class/meetings
    - Face to face during timetabled class(es)
    - Virtual learning environment

- Other (please specify)

16. How important does your institution consider promoting research skills among veterinary students?
    - Very important
    - Somewhat important
    - Neutral
    - Somewhat unimportant
    - Not important at all
17. In your view, what additional resources or structural changes would most enhance veterinary students' research involvement at your college? (Open-ended)
18. What is the total full time equivalent (FTE) of all faculty attributed to research at your college? (i.e., one full-time faculty member working 100% in research = 1 FTE. A faculty member working 50% in research = 0.5 FTE. Please estimate the total faculty FTE attributed to research at your college.) (numeric entry)
19. Do you feel that the total FTEs allotted to research is sufficient to provide opportunities for interested students to have access to research opportunities?
    - Yes
    - No
    - Maybe
20. If no, what do you feel that the minimum number of total faculty FTEs would need be to meet the students' needs at your institution? (numeric entry)
21. What percentage of faculty at your college mentor veterinary student researchers? (numeric entry)
22. Do you feel that this percentage of the faculty serving as mentors is sufficient for students wanting research mentors?
    - Yes
    - No
    - Maybe
23. If no, what do you feel that the minimum percentage of faculty serving as mentors would be to meet the needs of the students at your institution? (numeric entry)

**Support from Professional Bodies and Accreditors**

24. What, if anything, can professional organizations or accrediting bodies do to improve student participation in research? (Open-ended)

25. Should the Council On Education (COE) Standard 10 (the Research Standard) include specific policies or minimum expectations for students' participation in research? If so, what do you think these policies or minimum expectations should be, or how should they be developed? (Open-ended)

**Final Comments**

26. Please share any success stories, innovative initiatives, or lessons learned regarding veterinary student research engagement at your institution. (Open-ended)

27. Any other comments or suggestions? (Open-ended)
